# Time and tide of cerebellar synchrony

**DOI:** 10.1073/pnas.2204155119

**Published:** 2022-04-22

**Authors:** Chris I. De Zeeuw, Vincenzo Romano

**Affiliations:** ^a^Department of Neuroscience, Erasmus MC, 3015 GE Rotterdam, The Netherlands;; ^b^Netherlands Institute for Neuroscience, Royal Academy of Arts and Sciences, 1105 BA Amsterdam, The Netherlands

**Fig. 1.**
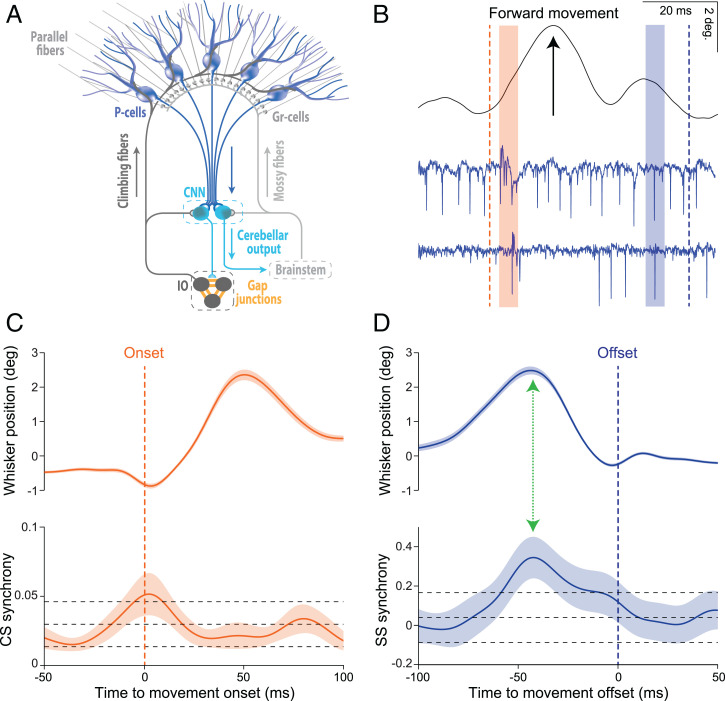
Synchrony of CSs and SSs may be correlated with onset and offset of movements, respectively. (*A*) Schematic representation of the convergence of inhibitory Purkinje cells (P-cells) onto cerebellar nuclei neurons (CNNs). P-cells integrate the inputs received via the climbing fiber and mossy fiber–parallel fiber systems and compute an output that can be read out by the CNNs. When the converging P-cell output is synchronous, the downstream effects at the CNNs may be subject to training and/or elicit sequences of relative silence and subsequent rebound activity. CNNs either directly control behavior via premotor areas in the brainstem or they provide feedback to neurons in the inferior olive (IO), which are coupled by gap junctions and provide the climbing fibers to the P-cells. Granular cells (Gr-cells) receive input from the mossy fibers and give rise to the parallel fibers. (*B*) Raw traces of a voluntary whisker movement (black line, top) and two simultaneously recorded PC-cells (blue traces, bottom). The orange and blue dashed lines indicate the onset and offset of the movement, respectively, while orange and blue beams indicate periods during which both PC-cells show CSs and SSs, respectively. (*C*) Average whisker movements (top) and synchrony of CS activity (bottom) centered around movement onset (dashed orange line). (*D*) Average whisker movements (top) and synchrony of SS activity (bottom) centered around movement offset (dashed blue line). CS synchrony rises just before movement onset and SS synchrony occurs before the whisker movements cease. Note that the peak of SS synchrony coincides at a moment of deceleration (green arrow), similar to the main finding by Sedaghat-Nejad et al. in PNAS (17). Top and bottom black dashed lines demarcate two SDs higher or lower than the mean of the synchrony levels as observed during the resting epoch (−200 to −150 ms prior to movement onset). CS and SS data are from 106 P-cell pairs from Romano et al. (18, 19). Note that whisker movements in *B*, *C*, and *D* are presented as position signals. Shaded areas indicate the SEM.

Just over half a century ago Bell and Grimm (1) were the first to record simultaneously from multiple Purkinje cells, revealing that different Purkinje cells can fire in synchrony within the same few milliseconds. This held true for both the complex spikes (CSs) that are modulated by the climbing fiber system and the simple spikes (SSs) that are modulated by the mossy fiber–parallel fiber system (Fig. 1 *A* and *B*). Since climbing fibers originate from neurons in the inferior olive that are extensively coupled by gap junctions (2, 3) and tend to oscillate (4, 5), systems physiologists have focused largely on the question of what the role of CS synchrony might be. To date, several studies over the past decades have revealed that CS synchrony may contribute to the coordination of motor behavior. For example, Welsh et al. (6) have provided compelling evidence that dynamic patterning of CS synchrony may allow for different combinations of muscles to be used to facilitate the timing and sequence of movements. Indeed, synchronized patterns of CS activity may contribute not only to the initiation of relatively simple reflex movements (7, 8) but also to more complex types of behaviors that require extensive training over time (9–12). Moreover, recent studies raise the possibility that CS signaling, and thereby CS synchrony, in particular microzones, might also be involved in reward signaling following acquisition of complex behaviors (9, 13, 14).

Unlike the progress in our understanding of the potential role(s) of synchronous firing of CSs, that of the SSs has been trailing behind. SS synchrony may increase with increasing CS synchrony (15), SS synchrony during movements may be greater among Purkinje cells that process the same type of signals (e.g., horizontal versus vertical eye movement signals) (15), and SS synchrony of Purkinje cells receiving input from the same parallel fiber beam may be correlated with certain movement epochs (16), but so far SS synchrony of single-unit Purkinje cells has not been directly correlated with any specific kinematic parameter. This long-standing lack of a novel concept backed up with empirical evidence is now provided by Sedaghat-Nejad et al. (17) in PNAS. They show that SS synchrony of Purkinje cells in the oculomotor vermis of marmosets can be associated with the end of targeted or spontaneous saccadic eye movements in that it peaks at the onset of deceleration. Importantly, this correlation cannot be biased by an increase in SS firing rate, because it actually drops. As such, the increase in synchrony of SSs diverges from that of CSs, which usually increases when the firing rate increases (7, 8). The probability for SS synchronization was greatest for saccades that were in the opposite direction of that for optimal CS modulation, elucidating the general association between SS synchrony and CS synchrony uncovered previously (15).

Inspired by the concept that SS synchrony may facilitate the timing of deceleration of movements, one can now go back to previously published datasets of Purkinje cell pairs related to other types of behaviors relevant for other species. For example, Romano et al. (18, 19) have recorded from Purkinje cell pairs in crus I and II during whisker movements in mice. In these datasets, one can observe a relation not only between CS synchrony and movement onset (Fig. 1*C*) but indeed also between SS synchrony and movement deceleration (Fig. 1*D*). Thus, the discovery by Sedaghat-Nejad et al. (17) may generalize across cerebellar lobules, types of behavior, and species.

How may SS synchrony exert its effects downstream in the cerebellar nuclei? Given that the synchrony is maximal during SS suppression (17), it may well exert its effects when the cerebellar nuclei neurons are disinhibited by reduced inhibitory input from the GABAergic Purkinje cells. Due to the modest Purkinje-to-nuclear convergence ratio and fast inhibitory postsynaptic current kinetics, the increased SS synchrony could result in a reaction of burst activity, made of spikes with very precise timing at the millisecond scale (20), via which premotor processes in the brainstem can be fine-tuned at a high temporal resolution (Fig. 1*A*). In other words, cerebellar nuclei neurons may be particularly sensitive for the level of SS synchrony during SS suppression and thereby able to control the timing of deceleration of movements. As highlighted in the paper by Sedaghat-Nejad et al. (17), this aligns indeed well with the profound impact of cerebellar cortical lesions on the termination of movements, including that of saccades (21).

By highlighting their concept on SS synchrony, Sedaghat-Nejad et al. (17) have opened up an avenue of new interesting research lines and corresponding questions. For example, their findings raise the intriguing possibility that whereas synchrony of CSs could facilitate the movement initiation, that of SSs may determine movement cessation (Fig. 1 *B*–*D*). Given that SS synchrony was highest between Purkinje cell pairs that showed optimal CS modulation around the same axis in space (albeit in opposite direction as the SS modulation), it appears likely that the start and end of a movement are efficiently coordinated within the same upbound or downbound module controlling particular muscle pairs (22). Likewise, the findings of Sedaghat-Nejad et al. (17) raise interesting questions about the relation between rate coding and temporal coding. Whereas the synchrony of SSs was highest at the deceleration of movements, the firing rate of SSs was quite different, often showing activation and suppression at the beginning and end of the movements, respectively. Given the relevance of rate coding for motor control by a population of Purkinje cells within a module (17), one wonders whether temporal coding of SSs is only relevant during a particular window of opportunity, i.e., when firing rate is low in the majority of the population involved. Finally, to what extent does learning play a role in generating SS synchrony and making it work downstream? On the one hand, one might hypothesize that it may not be necessary per se, as inhibitory molecular layer interneurons, which are coupled by gap junctions, appear well-suited to enhance both suppression and synchrony of SS activity within a particular microzone at the same time (22). This possibility is in line with the finding that SS synchrony did occur not only during saccades toward a particular target following training but also during spontaneous saccades (17). On the other hand, one might speculate that for SS synchrony to exert its effects downstream in the cerebellar nuclei, the nuclear cells might need to be entrained, requiring a learning process (20). Thus, the current work led by Reza Shadmehr (17) provides a wave of exciting new questions which may propagate in a timely manner on the tide of cerebellar synchrony.
